# A Synthetic Chloride Channel Relaxes Airway Smooth Muscle of the Rat

**DOI:** 10.1371/journal.pone.0045340

**Published:** 2012-09-26

**Authors:** Kwok-hei Yau, Judith Choi-wo Mak, Susan Wai-sum Leung, Dan Yang, Paul M. Vanhoutte

**Affiliations:** 1 Morningside Laboratory for Chemical Biology, Department of Chemistry, Faculty of Science, The University of Hong Kong, Hong Kong SAR, China; 2 Department of Pharmacology and Pharmacy, Li Ka Shing Faculty of Medicine, The University of Hong Kong, Hong Kong SAR, China; 3 Department of Medicine, Li Ka Shing Faculty of Medicine, The University of Hong Kong, Hong Kong SAR, China; The Chinese University of Hong Kong, Hong Kong

## Abstract

Synthetic ion channels may have potential therapeutic applications, provided they possess appropriate biological activities. The present study was designed to examine the ability of small molecule-based synthetic Cl^–^ channels to modulate airway smooth muscle responsiveness. Changes in isometric tension were measured in rat tracheal rings. Relaxations to the synthetic chloride channel SCC-1 were obtained during sustained contractions to KCl. The anion dependency of the effect of SCC-1 was evaluated by ion substitution experiments. The sensitivity to conventional Cl^–^ transport inhibitors was also tested. SCC-1 caused concentration-dependent relaxations during sustained contractions to potassium chloride. This relaxing effect was dependent on the presence of extracellular Cl^–^ and HCO_3_^−^. It was insensitive to conventional Cl^–^ channels/transport inhibitors that blocked the cystic fibrosis transmembrane conductance regulator and calcium-activated Cl^–^ channels. SCC-1 did not inhibit contractions induced by carbachol, endothelin-1, 5-hydroxytryptamine or the calcium ionophore A23187. SCC-1 relaxes airway smooth muscle during contractions evoked by depolarizing solutions. The Cl^–^ conductance conferred by this synthetic compound is distinct from the endogenous transport systems for chloride anions.

## Introduction

Synthetic ion channels are of interest because of their potential therapeutic and research applications. Although several synthetic ion channels have been synthesized and characterized [1]–[4], little information is available concerning their biological effects. Indeed, most of the characterization were carried out on abiotic systems. Typically, ion transport activities were studied using liposome-based fluorescence assays and channel activities were documented in planar lipid bilayer experiments [2]. Whether ion transport activity observed in these systems can be extrapolated to biological systems is, however, uncertain. A few studies have demonstrated the abilities of synthetic ion channels to kill bacteria [5]–[8] and to induce epithelial chloride (Cl^–^) secretion [9]–[12]. The action of synthetic ion channels on other biological systems, however, remains elusive. Previous studies have demonstrated the abilities of a small molecule-based synthetic Cl^–^ channel assemblage [13] to alter membrane potential, the intracellular calcium concentration ([Ca^2+^]_i_) and the contraction level in cultured vascular smooth muscle cells [14].

**Figure 1:**
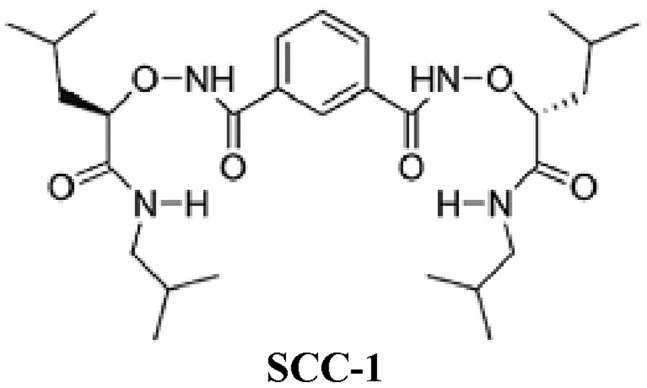
Chemical structure of SCC-1.

Ligand-gated Cl^–^ channels, γ-aminobutyric acid type A (GABA_A_) receptor, have been detected in both human and guinea pig airway smooth muscles [15]. In both guinea pig and human trachea, selective activation of these receptors *in vitro* reduces agonist-induced contractions [15], [16] and potentiates isoproterenol-mediated relaxation [16]. *In vivo*, selective GABA_A_ agonists attenuate agonist-induced airway constriction when central parasympathetic, postganglionic sympathetic, and inhibitory nonadrenergic, noncholinergic (NANC) neural contributions to airway tone are eliminated, suggesting a direct effect on the airway smooth muscle GABA_A_ receptor [17]. In addition, glycine-activated Cl^–^ channel (GlyR) are expressed in guinea pig and human airway smooth muscle [18]. Like that of GABA_A_ receptors, activation of GlyR relaxes agonist-contracted isolated airway smooth muscle and potentiates isoproterenol-mediated relaxation [18]. These observations prompted the investigation of biological activity of synthetic Cl^–^ channels in airway smooth muscle. Thus, the present study was designed to examine the effect of SCC-1 [a synthetic molecule-derived ion channel, designed in the laboratory [13], Fig. 1] on the responsiveness of the isolated trachea of the rat.

## Results

### SCC-1 Relaxed High-K^+^-induced Airway Smooth Muscles Contraction

**Figure 2:**
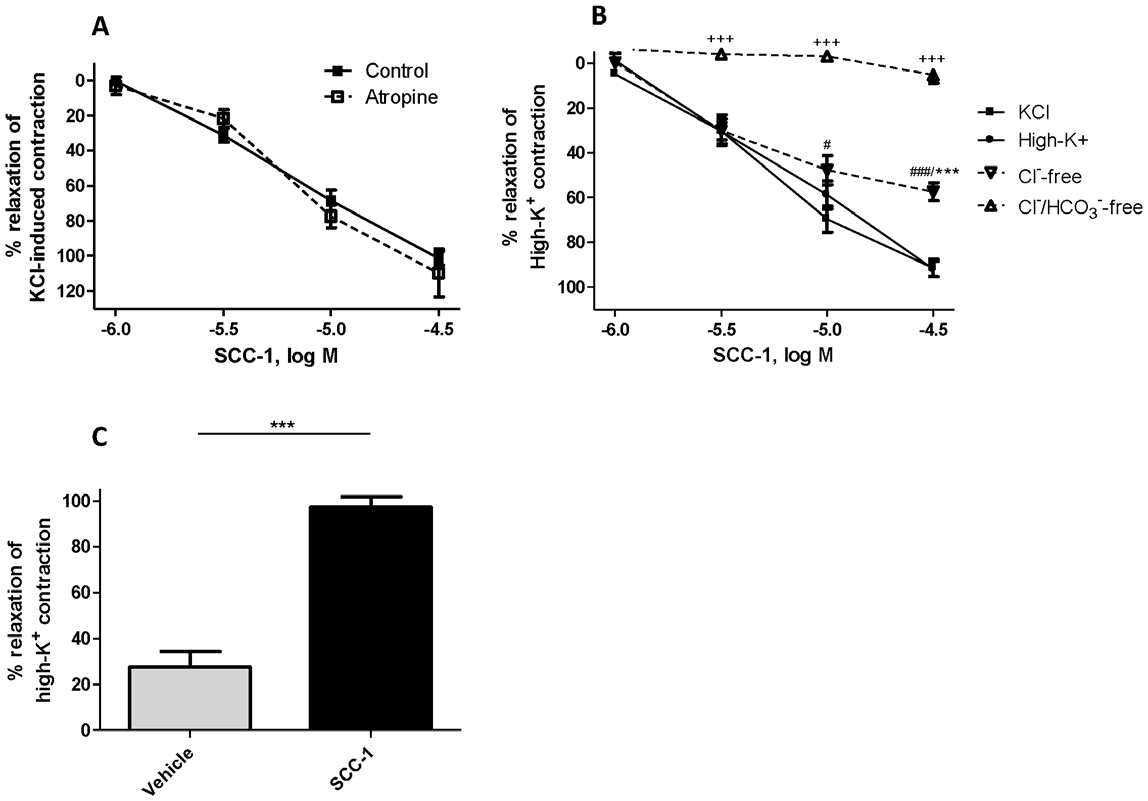
Effect of SCC-1 on high-K^+^-induced airway smooth muscle contraction. A) Concentration-response curves for the relaxant effect of SCC-1 on the rat tracheal rings contracted by 60 mM KCl in the absence (▪) or presence (□) of atropine (10^−6^ M). B) Concentration-response curves for the relaxing effect of SCC-1 in the rat tracheal rings contracted by 60 mM KCl (▪), high-K^+^ solution (•) and high-K^+^-Cl^–^-free solution (▽), high-K^+^-Cl^−/^HCO_3_^−^-free solution (△). */^#^ *p*<0.05, **/^##^ *p*<0.01, ***/^###/+++^ *p*<0.0001, two-way ANOVA followed by Bonferroni *post hoc* test. *, high-K^+^ versus high-K^+^-Cl^–^-free solution. ^#^, 60 mM KCl versus high-K^+^-Cl^–^-free solution, ^+^, high-K^+^-Cl^−/^HCO_3_^−^-free solution versus all groups. C) In the presence of 3×10^−5^ M of SCC-1 addition of 60 mM KCl to the organ chamber containing high-K^+^-Cl^−/^HCO_3_^−^-free solution caused relaxation of airway smooth muscles. ***p<0.0001, one-way ANOVA followed by Dunnett’s *post hoc* test. Data are presented as mean ± SEM, *n* = 4.

Addition of SCC-1 relaxed tracheal rings contracted with 60 mM KCl (Fig. 2A). This relaxation was concentration-dependent and when the SCC-1 concentration in the bath reached 3×10^−5^ M, the tension returned to the baseline level. Treating the tissues with muscarinic receptor antagonist atropine (10^−6^ M) did not affect the relaxing effects of SCC-1 **(** Fig. 2A **)**. The KCl-induced contraction was attenuated significantly by atropine (see Fig. S1).

Since the addition of KCl raised the osmolarity of the bath solution, whether this hyperosmolarity influenced the activity of SCC-1 was in question. The problem was addressed by monitoring the effect of SCC-1 on preparations contracted with iso-osmotic high-K^+^ solution and compared it to the hyperosmotic situation. The relaxing effects of SCC-1 were not significantly different in both cases (Fig. 2B). To investigate the Cl^–^ dependence of the action of SCC-1, high-K^+^-Cl^–^-free solution was used to obtain the background contraction. In the absence of extracellular Cl^–^, the effect of SCC-1 was attenuated significantly (Fig. 2B). In addition, the relaxing effect of SCC-1 on tracheae contracted by high-K^+^-Cl^−/^HCO_3_^−^-free solution was abolished (Fig. 2B). However, without washing out SCC-1, the addition of 60 mM KCl to the organ chamber in the presence of high-K^+^-Cl^−/^HCO_3_^−^-free solution caused relaxation (Fig. 2C).

**Figure 3:**
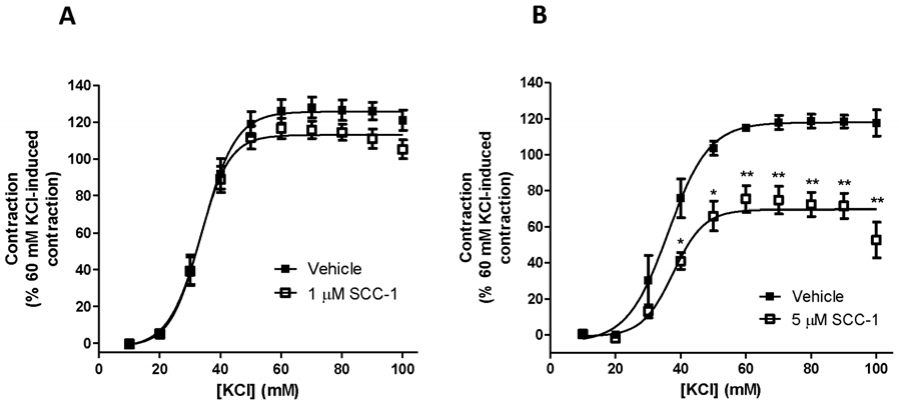
Contractile responses of isolated rat trachea rings to 60 mM KCl in the absence (▪) or presence (□) of SCC-1 (A an B, 1 and 5×10^−6^ M respectively). Contractions are expressed as a percentage of the response to 60 mM KCl. Data are presented as mean ± SEM, *n* = 4. *p<0.05, **p<0.01, Student’s *t*-test.

Alternatively, the effect of SCC-1 was assessed by treating the tissues prior to the cumulative addition of KCl. At 10^−6^ M, SCC-1 did not significantly affect the EC_50_ (Fig. 3A; vehicle control: 32.59±0.92 mM versus SCC-1∶29.54±1.415 mM; *p* = 0.12) but reduced the maximal contractile effect of KCl (vehicle control: 125.7±2.54% versus SCC-1∶112.9±2.28%; *p*<0.01). In the presence of SCC-1 (5×10^−6^ M), the maximal contraction evoked by KCl was reduced significantly (Fig. 3B; vehicle control: 118±2.77% versus SCC-1∶69.63±3.04%; *p*<0.0001) whereas the EC_50_ values were not altered significantly (vehicle control: 37.68±2.05 mM versus SCC-1∶36.18±1.33 mM; *p* = 0.55). The inhibitory effects on KCl-evoked contraction were more pronounced at 5×10^−6^ M than at 10^−6^ M.

### Cl^–^ Transport Inhibitors

**Figure 4:**
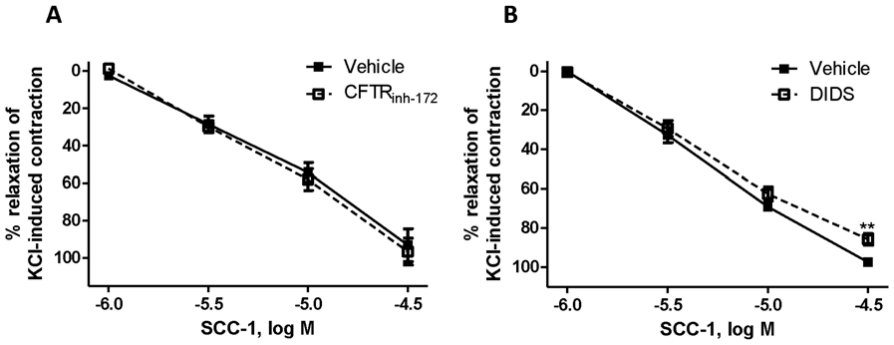
Concentration-response curves for the relaxing effect of SCC-1 (A and B) on the rat tracheal rings contracted by 60 mM KCl in the absence (▪) or presence (□) of Cl^–^-transport inhibitors. Data are presented as mean ± SEM, *n* = 4. **p<0.01, Student’s *t*-test.

The effects of three conventional Cl^–^ transport inhibitors, NPPB, DIDS and CFTR_inh-172_, on KCl-induced contraction were tested. NPPB (5×10^−5^ and 10^−4^ M) abolished KCl-induced contractions (see Fig. S2), preventing the use of that drug for further experiments. CFTR_inh-172_ at 10^−5^ M caused a modest but significant inhibition of the KCl-induced contraction, while DIDS at 10^−4^ M did not affect the response (see Fig. S2). At 10^−5^ M, CFTR_inh-172_ did not affect the relaxations to SCC-1, whereas DIDS (10^−4^ M) slightly but significantly reduced its effect at 3×10^−5^ M (Fig. 4A and 4B).

### Carbachol-, Endothelin-1-, 5-hydroxytryptamine- and A23187

**Figure 5:**
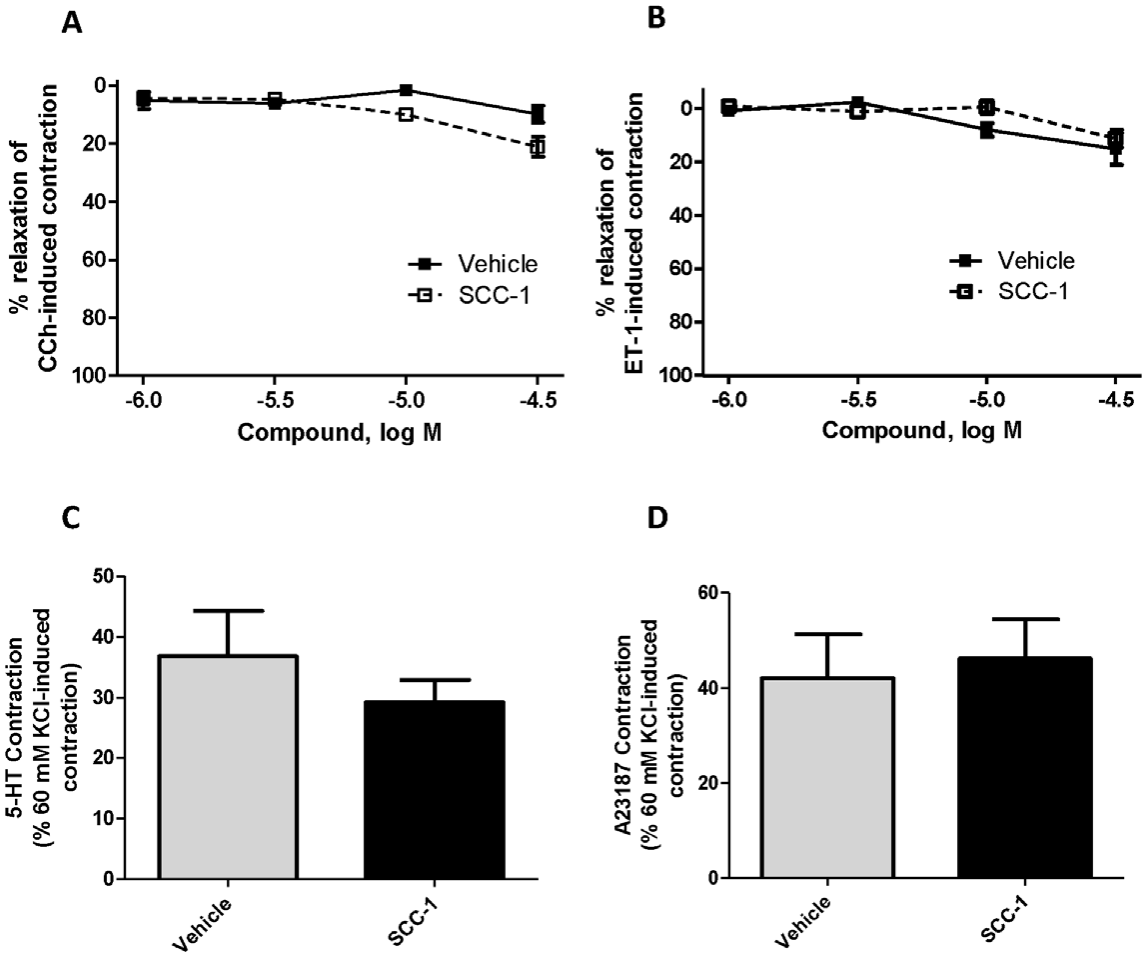
Effects of SCC-1 on contractions to carbachol (A), endothelin-1 (B), 5-HT (C) and A23187 (D). As both carbachol and endothelin-1 produced sustained contraction, SCC-1 was added to the bath in cumulative fashion when the plateau was reached. On the other hand, 5-hydroxytryptamine and A23187 induced transient contraction. The effect of the compounds were studied by incubating the tissues with SCC-1 (10^−5^ M) and the contraction evoked by 5- hydroxytryptamine and A23187 (both at 10^−6^ M) was subsequently monitored. Contractions are expressed as percentage of the response to 60 mM KCl. Data are presented as mean ± SEM, *n* = 4.

Contractions to either carbachol (10^−6^ M) or endothelin-1 (10^−7^ M) were not significantly influenced by the cumulative addition of SCC-1 (10^−6^−3×10^−5^ M) to the bath solution (Fig. 5A and 5B). Treating the tracheal rings with SCC-1 at 10^−5^ M had no effect on subsequent contractions evoked by 5-hydroxytryptamine or A23187 (both at 10^−6^ M) (Fig. 5C and 5D). The preparations did not contract in response to bradykinin, tachykinins or histamine (see Fig. S3) and therefore the effect of SCC-1 on the response to these agonists could not be evaluated.

## Discussion

The present study demonstrates that SCC-1 relaxes contractions of airway smooth muscle to high potassium in a Cl^–^-dependent manner. By contrast, contractions elicited by carbachol, endothelin-1, 5-hydroxytryptamine and A23187 are not affected by the synthetic Cl^–^ channel.

The effects of SCC-1 on the contraction of the trachea to high potassium are reminiscent of the observations in vascular smooth muscle [14]. Presumably, the relaxant effects of SCC-1 are related to their ability to form artificial Cl^–^ channels in cell membranes, which provide routes for transmembrane Cl^–^ movement [13]. The direction of Cl^–^ movement is dictated by the relative magnitude of membrane potential and the reversal potential for Cl^–^. Under the depolarizing conditions conferred by high-K^+^ -solution, the membrane potential is more positive than the reversal potential for Cl^–^ [19], and Cl^–^ channel formation would lead to Cl^–^ influx and hence membrane hyperpolarization. This clamps the membrane potential at a value more negative than the activation threshold for L-type Ca^2+^ channels [20], thereby limiting voltage-dependent Ca^2+^ influx and hence inhibition of KCl-induced contraction. Consistent with this interpretation, SCC-1 failed to relax contractions induced by the calcium ionophore A23187, suggesting that increases in [Ca^2+^]_i_ that is independent of activation of L-type Ca^2+^ channels were not affected. This latter finding also indicates that SCC-1 does not affect intracellular signaling downstream of the increased [Ca^2+^]_i_ leading to activation of the contractile proteins. As such, it is likely that SCC-1 relaxed the KCl-contracted airway smooth muscles by causing membrane hyperpolarization and deactivating L-type Ca^2+^ channels.

The present study demonstrated that KCl-induced contractions consisted in part of an atropine-sensitive cholinergic component and an atropine-resistant component attributed to direct Ca^2+^ influx *via* L-type Ca^2+^ channels. The full relaxation caused by SCC-1 implies that the synthetic compound may also antagonize the cholinergic component, which involves the release of acetylcholine by the presynaptic terminals, a process depending on Ca^2+^ influx *via* voltage-gated Ca^2+^ channels [21], followed by activation of postjunctional muscarinic M_3_ receptor [22]. As acetylcholine-induced contractions of airway smooth muscle are not blocked by L-type Ca^2+^ channels blocker [23], SCC-1 may affect the cholinergic component by inhibiting acetylcholine release from nerve terminals rather than by inhibiting the signaling pathway downstream of acetylcholine release. This interpretation was supported by the observation that SCC-1 did not affect carbachol-induced M_3_ receptor-mediated contraction.

The present study demonstrated that the effects of SCC-1 were independent of hyperosmolarity and the “high-Cl^–^-” condition caused by the addition of KCl to the bath. The presence of extracellular Cl^–^ was important for the activities of SCC-1 as indicated by the findings that: 1) eliminating Cl^–^ in the bath solution significantly attenuated the relaxing effects of SCC-1 and 2) while the latter failed to relax preparations contracted by high-K^+^-Cl^−/^HCO_3_^−^-free solution, the relaxing effect was restored after addition of 60 mM KCl. Moreover, the residual relaxing effects of SCC-1 during contractions to high-K^+^-Cl^–^-free solution were eliminated when HCO_3_^−^ was removed, suggesting that HCO_3_^−^ transport may also be involved in the actions of SCC-1. Likewise, CFTR, a natural Cl^–^ channel, has been suggested to increase HCO_3_^−^ permeability at low extracellular Cl^–^ levels [24] and to serve as HCO_3_^−^ channel [25].

The Cl^–^ conductance conferred by SCC-1 may be novel as its effects were insensitive to both CFTR_inh-172_ and DIDS, the conventional inhibitors of CFTR [26] and most non-CFTR Cl^–^ channels [27], respectively. In an earlier study, CFTR_inh-172_ and another Cl^–^ transport inhibitor DPC also did not affect the ability of SCC-1 to alter the membrane potential in Madin-Darby canine kidney **(**MDCK) cells [14]. Taken in conjunction, these findings suggest that SCC-1 forms synthetic Cl^–^ channels in the cell membranes of airway smooth muscle.

SCC-1 also failed to inhibit contractions elicited by carbachol, endothelin-1 and 5-hydroxytryptamine. These contractile agonists activate G_q_-coupled receptors, leading to the generation of multiple secondary messengers, including IP_3_ (which releases Ca^2+^ from intracellular stores), diacylglycerol, and activation of multiple Ca^2+^ channel types [28]. Bronchoconstrictors depolarize airway smooth muscle membrane [29]–[31], primarily by activation of Cl^–^ and non-selective cation currents as well as suppression of K^+^ currents [30], [31]. However, whether or not agonist-evoked contraction depends on Ca^2+^ influx *via* L-type Ca^2+^ channels remains controversial [32]. Indeed, some studies show that agonist-induced contraction of airway smooth muscles are not affected by L-type Ca^2+^ channels blockers [29], [33]. When the membrane potential was clamped at negative values below the activation threshold (–40 to –30 mV) for L-type Ca^2+^ channels, agonist-induced contractions are still observed [34]. Moreover, the normal range of membrane potentials (–70 to –30 mV) observed in airway smooth muscles [32], [35]–[40] is presumably too negative to activate L-type Ca^2+^ channels (−20 to +30 mV) [32], [41]–[44] and even at agonist concentrations reaching maximal contractile effects, the membrane potential is at a level that can only marginally activate L-type Ca^2+^ channels [32]. These studies prompt the suggestion that agonist stimulation is incapable of depolarizing the membrane to an extent that is sufficient to trigger significant voltage-dependent Ca^2+^ influx in airway smooth muscles. This conclusion is supported by the clinical findings that L-type Ca^2+^ channel blockers are relatively ineffective against asthma [45], [46]. If agonist-induced contractions of airway smooth muscle do not rely on voltage-dependent Ca^2+^ influx, modulation of voltage-dependent Ca^2+^ influx by synthetic Cl^–^ channels should not inhibit them.

In conclusion, the present study demonstrates the ability of SCC-1 to relax contracted airway smooth muscle. This relaxing effect partially depends on extracellular Cl^–^, consistent with the postulated Cl^–^ channel function conferred by SCC-1. The synthetic molecule-derived Cl^–^ conductance is novel because it is not inhibited by conventional Cl^–^ transport inhibitors. On the other hand, SCC-1 does not prevent agonist-induced contractions, which is explained by the voltage-independent nature of these responses.

## Materials and Methods

### Ethics Statement, Tissue Preparation and Isometric Tension Measurement

This investigation was approved by the Committee on the Use of Laboratory Animals for Teaching and Research of the University of Hong Kong. Adult male 12-weeks-old Sprague-Dawley rats (300–400 g) were maintained under a 12-h light/dark cycle at 21±1°C and were fed with standard laboratory chow (LabDiet 5053, USA) and tap water *ad libitum*. After being euthanized with pentobarbital sodium (70 mg/ml/kg, i.p.), their chest cavity was opened, and the lungs were removed *en bloc* and placed immediately into cold oxygenated Krebs-Henseleit solution of the following composition: 120 mM NaCl, 25 mM NaHCO_3_, 5.5 mM glucose, 4.76 mM KCl, 1.18 mM MgSO_4_·7H_2_O, 1.18 mM NaH_2_PO_4_·2H_2_O and 1.25 mM CaCl_2_·2H_2_O (control solution). The trachea was dissected free of connective tissues and fat and cut into rings (2–3 mm width). The rings were suspended between two stainless steel hooks in organ chambers filled with 5 mL of control solution. One of the hooks was attached to the organ chamber and the other was connected to a force transducer (AD Instruments, model MLT0201/D, Bella Vista, Australia) for isometric force recording. The bathing solution was maintained at 37°C and continuously aerated with a mixture of 95% O_2_ and 5% CO_2_. The rings were allowed to equilibrate under 1 g of tension for 60 min with bathing solution changes every 15 min. During the equilibration, the tension was adjusted to 1 g, except for the last 30 min.

High-K^+^ (64.76 mM K^+^) solution was prepared by iso-osmotic replacement of NaCl with equimolar amounts of KCl. For the high-K^+^-Cl^–^free solution, equimolar Na-gluconate replaced NaCl, K-gluconate replaced KCl and 5 mM Ca-gluconate replaced CaCl_2_. Calcium was increased to 5 mM to compensate for the Ca^2+^- buffering capacity of gluconate. The high-K^+^-Cl^−/^HCO_3_^−^-free solution has the following composition: 60 mM Na-gluconate, 64.76 mM K-gluconate, 20 mM Ca-gluconate, 19.5 mM D-mannitol, 5.5 mM glucose, 1.18 mM MgSO_4_·7H_2_O, 1.18 mM NaH_2_PO_4_·2H_2_O, 5.6 mM tris and 10 mM HEPES. The solution was gassed with 100% O_2_.

### Effect of SCC-1 on Contractions

Each tracheal ring was contracted with the agonist at the indicated concentration and was allowed to achieve a steady-state plateau. SCC-1 was then added to the bath in a cumulative fashion (10^−6^–3×10^−5^ M). This protocol was used for contractile agents that induced sustained contraction, namely, KCl, high-K^+^ solution, high-K^+^-Cl^–^-free solution, high-K^+^-Cl^−/^HCO_3_^−^-free solution, carbachol and endothelin-1. For the contraction induced by high-K^+^-Cl^−/^HCO_3_^−^-free solution, when the SCC-1 concentration in the bath reached 3×10^−5^ M and a steady-state response was obtained, 60 mM KCl was added to the baths to test the Cl^–^-dependency of the effects of SCC-1.

Alternatively, the effect of the synthetic ion channels was determined by adding a single concentration of the compound to the organ chamber 30 min before the addition of contractile agonists and observing how the airway smooth muscle contractility was altered. In this case, the concentration of the tested compound was maintained throughout the experiment. This protocol was primarily used for agonists that induced transient contraction, namely 5-hydroxytryptamine and A23187.

### Cl^–^ Transport Inhibitors

The effect of Cl^–^ transport inhibitors (10^−5^ M CFTR_inh-172_, 10^−4^ M DIDS, 5×10^−5^ and 10^−4^ M NPPB) on KCl-evoked contraction was evaluated by adding the inhibitors at the indicated concentrations to the bath 30 min before the addition of 60 mM KCl and observing how the responsiveness of the airway smooth muscle was altered. The inhibitor concentration was maintained throughout the experiment. When steady-state contraction was achieved, SCC-1 was added to the organ chamber in a cumulative fashion (10^−6^–3×10^−5^ M).

### Chemicals and Drugs

4,4′-Diisothiocyanato-stilbene-2,2′-disulphonic acid (DIDS), 5-hydroxytryptamine (5-HT), the calcium ionophore A23187, carbachol and endothelin-1 were purchased from Sigma Chemicals Co. (St. Louis, MO, USA); CFTR_inh-172_ from Calbiochem (San Diego, CA, USA) and atropine sulfate from Merck (Darmstadt, Germany). Stock solutions of N1,N3-bis((R)-1-(isobutylamino)-4-methyl-1-oxopentan-2-yloxy)isophthalamide, or SSC-1 (synthesized in the laboratory, Fig. 1), A23187, CFTR_inh-172_ and DIDS stock were prepared in dimethylsulfoxide (DMSO) while all stock solutions of the remaining drugs were dissolved in de-ionized water.

### Data and Statistical Analysis

Data are presented as means ± standard error of the means (SEM), and *n* indicates the number of rats used in the experiments. Changes in tension were expressed as a percentage of the reference contraction in response to the addition of KCl (60 mM). Relaxation to SCC-1 was expressed as percent reduction of active force (the difference between baseline tension and peak tension generated by contractile agonists). For concentration-response studies, the data were fit by non-linear regression analysis where appropriate. Maximal contractions and EC_50_ (the concentration of agonist required to produce 50% of the maximal response) were determined. Statistical analysis was performed using two-tailed unpaired Student’s t-tests or ANOVA followed by Bonferroni’s or Dunnett’s *post hoc* comparison as appropriate. All statistical procedures were computed using Prism version 5.01 (GraphPad Software, San Diego, CA, USA). P-values less than 0.05 were considered to indicate statistically significant differences.

## Supporting Information

Figure S1 **Contractile responses of isolated rat trachea rings to KCl (A) in the absence (▪) or presence (□) of atropine (10^−6^ M). Incubating the tissues with atropine (10^−6^ M) attenuated the contractile effect of KCl (A, open squares).** Contractions are expressed as a percentage of the response to 60 mM KCl. Data are presented as mean ± SE, *n* = 6. *p<0.05, **p<0.01, ***p<0.0001, Student’s *t*-test.
(TIF)

Figure S2 **The effects of conventional Cl^–^-transport inhibitors on airway smooth muscles contraction evoked by 60 mM KCl.** Contractions are expressed as a percentage of the response to 60 mM KCl. Data are presented as mean ± SE, *n* = 4. *p<0.05, ***p<0.0001, Student’s *t*-test.
(TIF)

Figure S3 **Contractile responses of isolated rat trachea rings to bradykinin (A), endothelin-1 (B) and histamine (C).** Contractions are expressed as a percentage of the response to 60 mM KCl. Data are presented as mean ± SE, n = 4.
(TIF)
