# Supplementary figures and images for: A Synthetic Chloride Channel Relaxes Airway Smooth Muscle of the Rat
Source: PLoS One. 2012 Sep 26;7(9):e45340. doi: 10.1371/journal.pone.0045340 (PMC3458840; doi:10.1371/journal.pone.0045340)

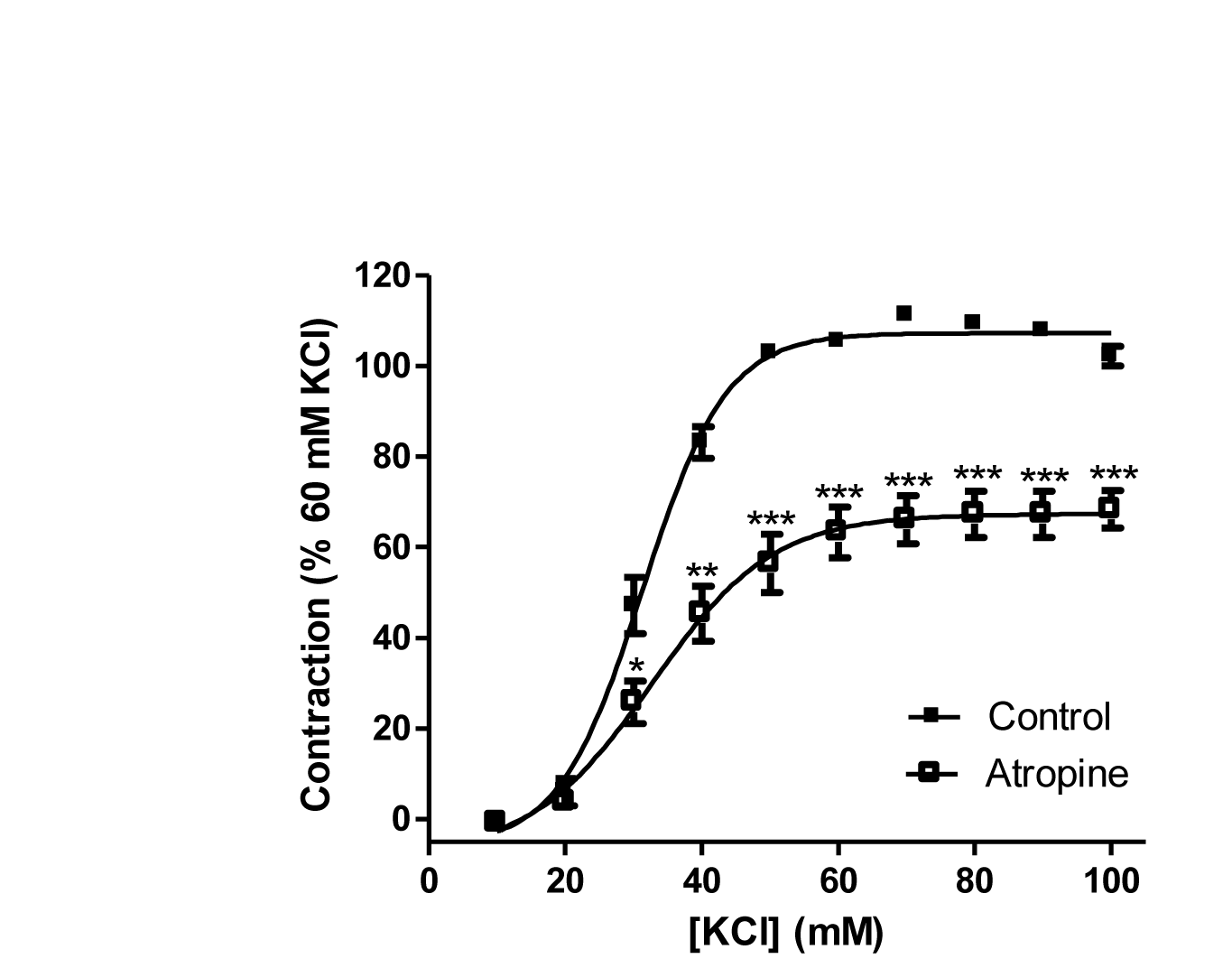

Supplement: Figure S1 — Contractile responses of isolated rat trachea rings to KCl (A) in the absence (▪) or presence (□) of atropine (10−6 M). Incubating the tissues with atropine (10−6 M) attenuated the contractile effect of KCl (A, open squares). Contractions are expressed as a percentage of the response to 60 mM KCl. Data are presented as mean ± SE, n = 6. *p<0.05, **p<0.01, ***p<0.0001, Student’s t-test. (TIF) [file pone.0045340.s001.tif]

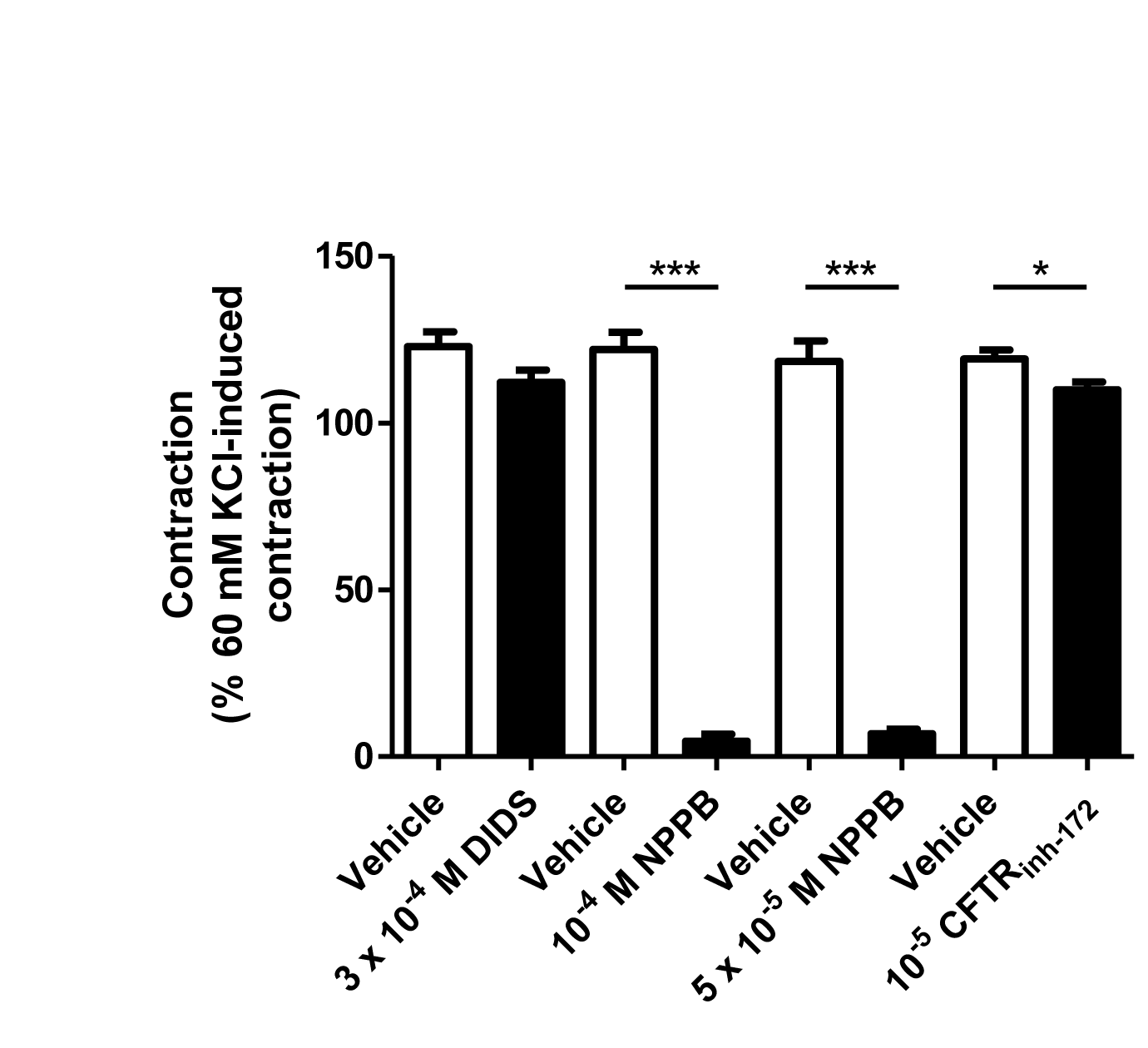

Supplement: Figure S2 — The effects of conventional Cl–-transport inhibitors on airway smooth muscles contraction evoked by 60 mM KCl. Contractions are expressed as a percentage of the response to 60 mM KCl. Data are presented as mean ± SE, n = 4. *p<0.05, ***p<0.0001, Student’s t-test. (TIF) [file pone.0045340.s002.tif]

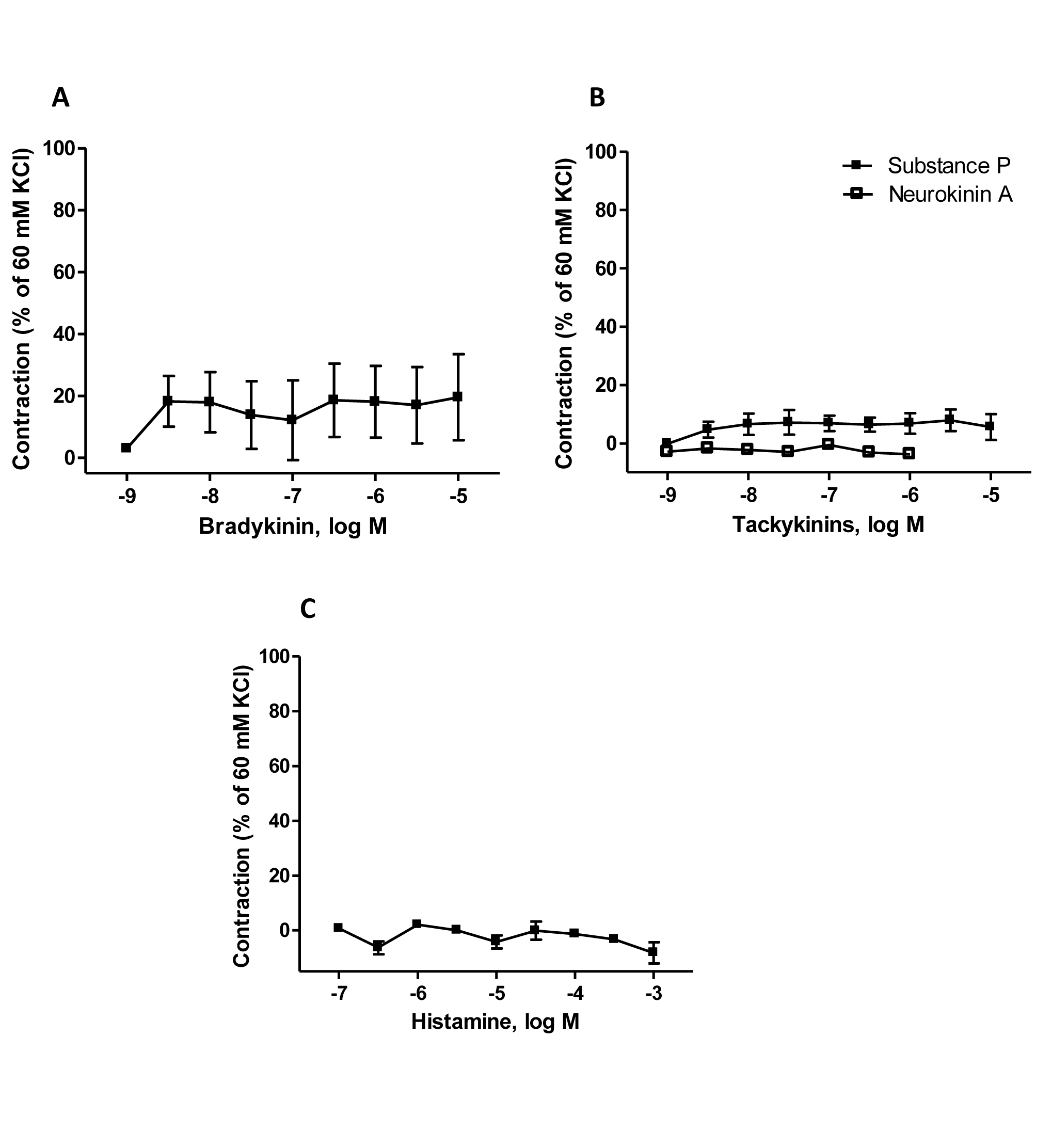

Supplement: Figure S3 — Contractile responses of isolated rat trachea rings to bradykinin (A), endothelin-1 (B) and histamine (C). Contractions are expressed as a percentage of the response to 60 mM KCl. Data are presented as mean ± SE, n = 4. (TIF) [file pone.0045340.s003.tif]
